# Role of miR-182 in cardiovascular and cerebrovascular diseases

**DOI:** 10.3389/fcell.2023.1181515

**Published:** 2023-05-09

**Authors:** Gaiqin Pei, Li Chen, Yang Wang, Chengqi He, Chenying Fu, Quan Wei

**Affiliations:** ^1^ Department of Rehabilitation Medicine and Institute of Rehabilitation Medicine, West China Hospital, Sichuan University, Chengdu, Sichuan, China; ^2^ Key Laboratory of Rehabilitation Medicine in Sichuan Province, Chengdu, Sichuan, China; ^3^ Department of Rehabilitation, Chengdu Fifth People’s Hospital, Chengdu, Sichuan, China; ^4^ National Clinical Research Center for Geriatrics, West China Hospital, Sichuan University, Chengdu, Sichuan, China; ^5^ Aging and Geriatric Mechanism Laboratory, West China Hospital, Sichuan University, Chengdu, Sichuan, China

**Keywords:** cardiovascular and cerebrovascular diseases, miR-182, atherosclerosis, myocardial ischemia, ischemic stroke, heart failure, mesenchymal stromal cells, ischemia/reperfusion (I/R) injury

## Abstract

The treatment of cardiovascular and cerebrovascular diseases have undergone major advances in recent decades, allowing for a more effective prevention of cardiovascular and cerebrovascular events. However, cardiac and cerebral atherothrombotic complications still account for substantial morbidity and mortality worldwide. Novel therapeutic strategies are critical to improve patient outcomes following cardiovascular diseases. miRNAs are small non-coding RNAs, that regulate gene expression. Here, we discuss the role of miR-182 in regulating myocardial proliferation, migration, hypoxia, ischemia, apoptosis and hypertrophy in atherosclerosis, CAD, MI, I/R injury, organ transplant, cardiac hypertrophy, hypertension, heart failure, congenital heart disease and cardiotoxicity. Besides, we also summarize the current progress of miR-182 therapeutics in clinical development and discuss challenges that will need to be overcome to enter the clinic for patients with cardiac disease.

## Introduction

An estimated 17.9 million people died from cardiovascular diseases (CVDs) in 2019, accounting for 32% of all global deaths. Among these deaths, 85% were due to heart attack and stroke (WHO, 2019). CVDs are disorders of the heart and blood vessels including coronary heart diseases, cerebrovascular diseases and other conditions. Early detection of CVDs is crucial to reduce the mortality rate associated with cardiovascular diseases. Despite the fact that early revascularization therapy and drug treatment promote cardiac repair after ischemia and improve the survival rate of individuals with myocardial infarction (MI), some patients still experience heart failure (HF) (Parikh et al., 2009). Identifying factors for more effective prevention and early diagnosis remains a challenge. The mechanism and risk factors that intervene in disease occurrence and development need to be better explored.

miRNAs are single stranded, small (approx. 22 nucleotides), and endogenous non-coding RNA (ncRNA) molecules (Kim et al., 2009), which bind to the 3′-UTR of target messenger RNAs (mRNAs) causing the suppression of gene expression at both the mRNA and translational levels (Bartel, 2004). The identification of miRNAs has opened a new window to an important area of biology that was previously unexplored, but it also has important implications in human development and disease. miRNAs are active participants in a broad array of physiological homeostasis and pathological statuses (Saliminejad et al., 2019) (Rupaimoole and Slack, 2017). It has been estimated that miRNAs may be involved in the regulation of 30% of the protein-coding genes in the human genome (Sasso et al., 2022). miRNA-based regulation plays an important role in some crucial biological and cellular processes, such as differentiation, development, proliferation, apoptosis, metabolism and signal transduction pathways (Osman, 2012). However, a single miRNA can bind to hundreds of target mRNAs depending on target specificity and individual mRNAs can also be regulated by many different miRNAs (Bartel, 2004) (Fabian et al., 2010). Thus, their mechanism of action varies depending on the type of organs and cells, and sometimes even depending on the different stages of the disease (Winbanks et al., 2014) (Hata, 2013) (McClelland and Kantharidis, 2014). The multifunctionality of miRNAs has been extensively investigated in a range of diseases, including cardiovascular and cerebrovascular disorders (Beermann et al., 2016) (Lee and Dutta, 2009) (Ono et al., 2011).

**FIGURE 1:**
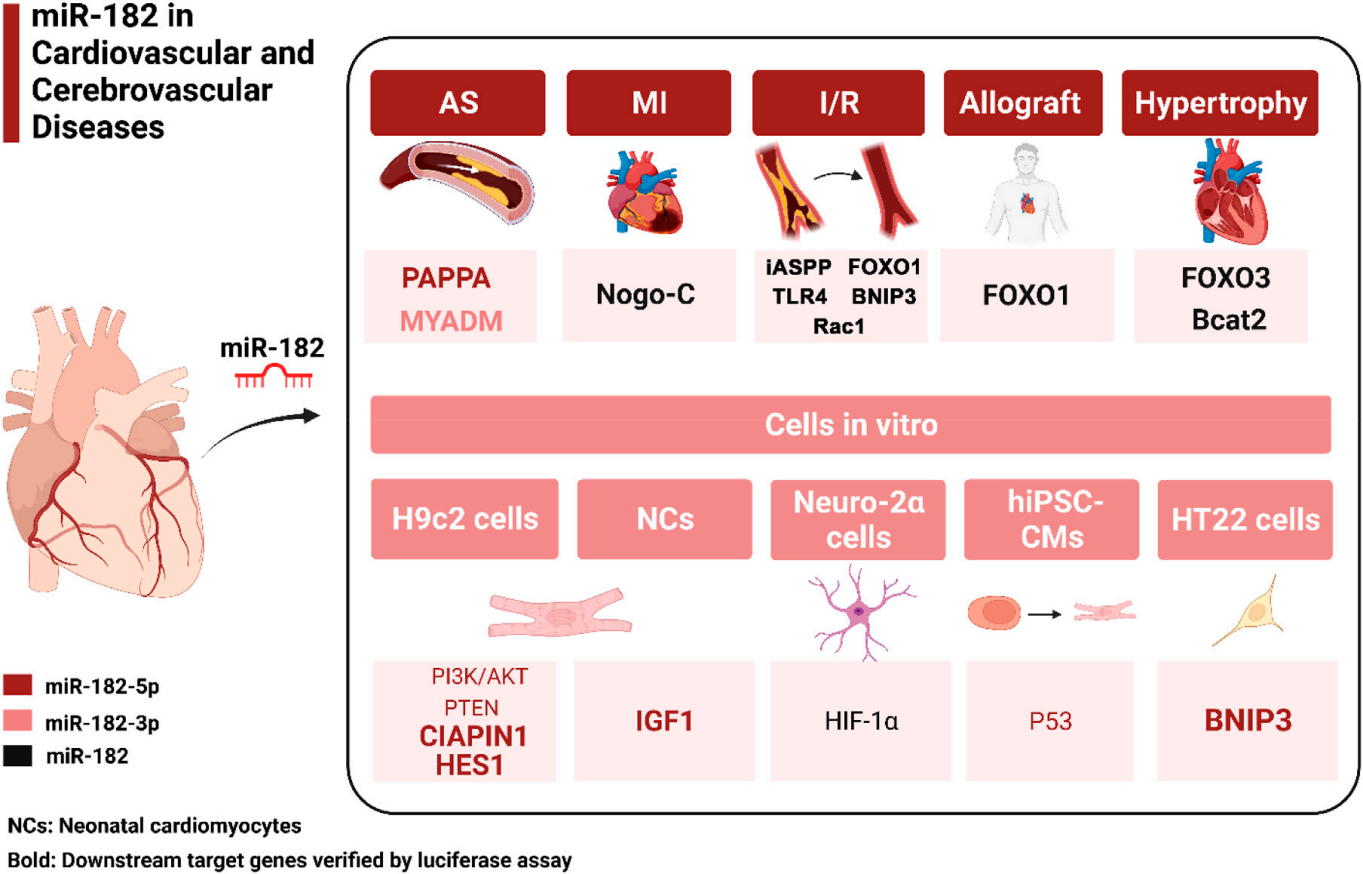
The target genes of miR-182 in different pathogenesis of cardiovascular and cerebrovascular diseases. Red, pink and black of downstream target genes represent the known miRNA-mRNA associations from miR-182-5p, miR-182-3p and miR-182, respectively. Bold indicates downstream target genes verified by luciferase assay. AS, atherosclerosis; MI, myocardial infarction; I/R, ischemia/reperfusion; PAPPA, pregnancy associated plasma protein A; MYADM, myeloid-associated differentiation marker; BNIP3, BCL2 Interacting Protein three; TLR4,Toll-like receptor four; FOXO1/3, forkhead box protein O1/3, CIAPIN1, cytokine-induced apoptosis inhibitor one; HES1, hairy and enhancer of split-1; iASPP: inhibitory member of the ASPP family; IGF1, insulin-like growth factor 1; HIF-1α, hypoxia-inducible factor; NCs, neonatal cardiomyocytes; hiPSC-CMs, human-induced pluripotent stem cell-derived cardiomyocytes; HT22, mouse hippocampal neuronal cells.

miRNAs can be grouped into families based on sequence conservation within the seed region (nucleotides 2–8) but may on different chromosomes (Bernardo et al., 2012). microRNA-182 (miR-182) is a miRNA that belongs to the miR-183/96/182 cluster and highly conserved in vertebrates. They are located within genes positioned within a short distance (∼10 kb) on human chromosome 7q31-34 and mouse chromosome 6q A3, which are generated from a single primary transcript and have similar expression patterns (Xu et al., 2007). miR-182 is composed of two homologs miR-182-3p and miR-182-5p. It is highly expressed in mouse retina and inner ear hair cells, which has been identified in various cells such as osteoblasts, lymphocytes, and adipocyte (Stittrich et al., 2010) (Kim et al., 2012) (Friedman et al., 2009) (Dong et al., 2020). Currently, miR-182 is closely associated with the normal differentiation of sensory organs, the immune system, lymphatic vessels, bone, memory formation, the development of tumors, autoimmune diseases, depression, and metabolic diseases (Friedman et al., 2009; Dai et al., 2010; Saus et al., 2010; Stittrich et al., 2010; Kim et al., 2012; Yu et al., 2012; Griggs et al., 2013; Zhang et al., 2013; Kiesow et al., 2015). And particularly, upregulated blood levels of miR-182 have been shown to be associated with prognosis in patients with chronic heart failure (Cakmak et al., 2015). New nano system which can efficiently deliver inhibitor of miR-182 into hearts can significantly suppress cardiac hypertrophy (Zhi et al., 2019). Additionally, the regulatory connections of ncRNAs, represented by miRNAs, long non-coding RNAs (lncRNAs), and circular RNAs (circRNAs), have been widely demonstrated to be involved in the regulation of gene expression and to affect multiple biological processes in cardiovascular and cerebrovascular diseases. Some ncRNAs, such as lncRNA SNHG16 (Wang et al., 2020), lncRNA UCA1 (Chen et al., 2022), circRNA_0008028 (Shi et al., 2022), and circ_002664 (Liu et al., 2020), have been reported to act as significant regulators in cardiovascular and cerebrovascular diseases by targeting miR-182. Some recent studies have also described the role of miR-182 in cardiovascular and cerebrovascular diseases (see Table 1). Therefore, this review primarily focuses on miR-182 to discuss its role in cardiovascular and cerebrovascular diseases, and to explore the possibility of miR-182 as a potential diagnostic and therapeutic target for clinical treatment (Figure 1).

**TABLE 1:** A tabulated summary of miR-182 involved in cardiovascular and cerebrovascular diseases.

References Country	miRNAExpression	Target genes	Signaling pathway	Target cells/tissues/organs	Disease or phenotype	Intervention	Experimental setting	Species
Jin 2020	miR-182–5p	PAPPA	IGF	HA-VSMC	CAD	Ox-LDL	*In vitro*	Human
			NF-kB					
China ^(Jin et al., 2020)^	↓		PI3K/AKT ERK				*In vivo*	
Sun 2016	miR-182–3p	MYADM	ERK/MAP	Human aortic artery SMCs	Proliferation	ADMA	*In vitro*	Rat Human
China ^(Sun et al., 2016)^	↓				Migration		*In vivo*	
Bai 2021	miR-182	MYADM	BMP/SMAD1/5/8	Human PA- SMCs/ECs	PAH	Hypoxia	*In vitro*	Rat Human
					Proliferation			
China ^(Bai et al., 2021)^			TGF-β/SMAD2/3		Migration	TGF-β	*In vivo*	
Sun 2020	miR-182–3p	MYADM	KLF4/p21	PASMCs	PAH	MCT	*In vitro*	Rat Human
China ^(Sun et al., 2020)^							*In vivo*	
Rancan 2016	miR-182	N	N	Lung biopsies	Lung I/RI	Lung autotrans-plantation	*In vitro*	Pig
Spain ^(Rancan et al., 2016)^	↑							
Li 2021	miR-182	XBP1	SIRT1/NLRP3	NCTC1469 cells hepatocyte	Hepatic I/R	H/R	*In vitro*	Mice
China ^(Li et al., 2021)^							*In vivo*	
Chaudhari 2016	miR-182–5p	N	N	hiPSC-CMs	Cardiotoxicity	Doxorubicin	*In vitro*	Human
Germany ^(Chaudhari et al., 2016)^	↑							
Gryshkova2022	miR-182–5p	N	N	hiPSC-CMs	Cardiotoxicity	Anthracyclines	*In vitro*	Human
UK ^(Gryshkova et al., 2022)^	↑							
Taurino 2010	miR-182	N	N	Plasma	CAD	N	*In vitro*	Human
UK ^(Taurino et al., 2010)^	↑							
Li 2020	miR-182–5p	N	N	N	Hypertension	N	WGCNA	N
China ^(Li et al., 2020)^	↑							
Zhu 2019	miR-182–5p	N	N	Plasma	uLMCAD	N	*In vitro*	Human
China ^(Zhu et al., 2019)^	↑							
Nováková 2019	miR-182–5p	N	N	Endomyocardial biopsies	ACR of heart allografts	N	*In vitro*	Human
Czech Republic^(Nováková et al., 2019)^	↓							
Wei 2012	miR-182	FOXO1	CD3+T cell	GILs Cardiomyocytes	Heart allograft rejection	N	*In vitro*	Mice
US ^(Wei et al., 2012)^	↑			Plasma			*In vivo*	
Wei 2017	miR-182	FOXO1	CD4+T cell	Heart Splenocytes PBMC GILs	Heart allograft rejection	CTLA4-Ig	*In vitro*	Mice
US ^(Wei et al., 2017)^							*In vivo*	
Fang 2022	miR-182–5p	N	N	Plasma	CHF	N	*In vitro*	Human
China ^(Fang et al., 2022)^	↑							
Cakmak 2015	miR-182	N	N	Plasma	CHF	N	*In vitro*	Human
Turkey ^(Cakmak et al., 2015)^								
Jia 2016	miR-182	Nogo-C	N	Heart	MI	CoCl2	*In vitro*	Rat
China ^(Jia et al., 2016)^	↓			NRCMs	Apoptosis		*In vivo*	
Zhang 2018	miR-182–5p	CIAPIN1	N	H9c2	MI	Hypoxia	*In vitro*	Rat
China ^(Zhang et al., 2018b)^	↑				Apoptosis			
Cui 2013	miR-182	N	N	Brain	HIBI	MI	*In vivo*	Rat
China ^(Cui and Yang, 2013)^	↓							
Zhao 2019	miR-182	TLR4	TLR4/NF-κB/PI3K/Akt	MSC-Exo RAW264.7 cells	I/R	LPS	*In vitro*	Mice
China ^(Zhao et al., 2019)^	↑				Macrophage polarization			
Alhadidi 2022	miR-182	N	Cortactin-Arp2/3	Astrocytes	Stroke	tMCAO	*In vitro*	Mice
					Cerebral I/R			
USA ^(Alhadidi et al., 2022)^	↑			Brain	Inflammation	OGD/R	*In vivo*	
Zhang 2020	miR-182	FOXO1	mTOR	bEnd.3 cells	Cerebral ischemia	pMCAO	*In vitro*	Mice
China ^(Zhang et al., 2020)^	↑			Brain	Apoptosis	OGD	*In vivo*	
Yi 2017	miR-182	iASPP	N	Neuro-2a cells	Cerebral ischemia	pMCAO	*In vitro*	Rat
China ^(Yi et al., 2017)^	↑			Brain	Oxidative stress	H_2_O_2_	*In vivo*	
Wang 2018	miR-182–5p	TLR4	N	BV2 cells	Cerebral ischemia	tMCAO	*In vitro*	Rat
China ^(Wang et al., 2018a)^	↓				Inflammation	OGD	*In vivo*	
Qi 2020	miR-182	N	N	Plasma	Stroke	N	*In vivo*	Human
China ^(Qi et al., 2020)^	↑							
Ding 2023	miR-182–5p	Rac1	N	Astrocytes	Cerebral I/R	tMCAO	*In vitro*	Mice
China ^(Ding et al., 2023)^	↑			Brain	Inflammation	OGD/R	*In vivo*	
Hu 2021	miR-182–5p	TLR4	MALAT1/miR-182–5p/TLR4	PC12 cells	Cerebral I/R	tMCAO	*In vitro*	Mice
China ^(Hu et al., 2021)^	↑			Brain	Inflammation	H/R	*In vivo*	
Lee 2010	miR-182	N	N	Ischemic cortex Neuro-2a cells	Cerebral ischemia	CAO	*In vitro*	Rat
South Korea ^(Lee et al., 2010)^	↑					OGD	*In vivo*	
Deng 2020	miR-182–5p	BINP3	N	HT22	Cerebral I/R apoptosis	OGD/R	*In vitro*	Mice
China ^(Deng et al., 2020)^	↓							
Huang 2020	miR-182	N	N	Heart	Cardiotoxicity	Trichloroethy-lene	*In vivo*	Zebrafish
China ^(Huang et al., 2020)^	↑							
Zhang 2018	miR-182	HES1	N	H9c2	CHD	Hypoxia	*In vitro*	Rat
China ^(Zhang et al., 2018c)^	↓				Cell proliferation Apoptosis			
Li 2016	miR-182	Bcat2	Akt/mTORC1	Heart	Cardiac hypertrophy Angiogenesis	PlGF	*In vitro*	Mice, Rat
China ^(Li et al., 2016)^	↑			NRCs			*In vivo*	
Wang 2020	miR-182–5p	IGF1	CTCF/SNHG16	NCs	Cardiac hypertrophy	Angiotensin II	*In vitro*	Mice
China ^(Wang et al., 2020)^	↓							
Zhi 2019	miR-182	N	N	Heart	Cardiac hypertrophy	Aortic coarctation	*In vitro*	Mice
China ^(Zhi et al., 2019)^				H2c9			*In vivo*	
Lee 2016	miR-182	BNIP3	N	Heart NRVCs	MI	MI	*In vitro*	Rat
South Korea ^(Lee et al., 2016)^	↑				Apoptosis Fibrosis	Hypoxia	*In vivo*	
Nemade 2018	miR-182–5p	N	N	hPSC-CMs	Cardiotoxicity	Etoposide	*In vitro*	Human
Germany ^(Nemade et al., 2018)^	↑							
Zhang 2018	miR-182–5p	N	GAA/PTEN/PI3K/AKT	H9c2	MI	Hypoxia	*In vitro*	Rat
China ^(Zhang et al., 2018a)^	↑							
Wang 2018	miR-182	N	N	Heart	Spontaneously hypertension	N	*In vivo*	Rat
China ^(Wang et al., 2018b)^	↑							
Ikitimur 2015	miR-182	N	N	Plasma	Systolic HF	N	*In vivo*	Human
Turkey ^(Ikitimur et al., 2015)^					LVMI			
Zhirov 2019	miR-182	N	N	Plasma	Acute decompensation of CHF	N	*In vivo*	Human
Russia ^(Zhirov et al., 2019)^	↑							

ACR: acute cellular rejection; ADMA: asymmetrical dimethylarginine; bEnd.3 cells: brain microvascular endothelial cell line; CAD: coronary artery disease; CAO: cerebral artery occlusion; CHF: chronic heart failure; FOXO: forkhead box proteins; GAA: Ganoderic acid A; GILs: Graft infiltrating lymphocytes; H/R: Hypoxia/reoxygenation; hiPSC-CMs: Human-induced pluripotent stem cell-derived cardiomyocytes; HIBI: hypoxic-ischemic brain injury; I/R: Ischemia/reperfusion; iASPP: inhibitory member of the ASPP, family; LCCA: left common carotid artery; LVMI: left ventricular mass index; t/p MCAO: transient/permanent middle cerebral artery occlusion; MI: myocardial infarction; MYADM: Myeloid-Associated differentiation marker; MCT: monocrotaline; MSC: mesenchymal stromal cell; NCs: Neonatal cardiomyocytes; NRCs: Neonatal rat cardiomyocytes; NRVCs: Neonatal rat ventricular cardiomyocytes; NRCMs: Neonatal rat cardiomyocytes; OGD: oxygen glucose deprivation; Ox-LDL: Oxidized low-density lipoprotein; PAH: pulmonary artery hypertension; PASMCs: Pulmonary arterial smooth muscle cells; PBMC: peripheral blood mononuclear cells; PlGF: placental growth factor; TAC: transversal aortic constriction; uLMCAD: unprotected left main coronary artery disease; VSMC: vascular smooth muscle cell; WGCNA: weighted gene coexpression network analysis.

### miR-182 in atherosclerosis and coronary artery disease

Coronary artery disease (CAD) caused by atherosclerosis (AS) is a leading cause of death and morbidity worldwide (Knuuti et al., 2019) (Frostegård, 2013). The proliferation, apoptosis, migration, and differentiation of vascular smooth muscle cells (VSMCs) play crucial roles in the pathogenesis of AS (Bennett et al., 2016). The role of oxidized low-density lipoprotein (ox-LDL) in the formation of foam cells derived from VSMCs, which act as one of the main risk factors for AS, has been extensively studied (Kattoor et al., 2019). In addition, asymmetrical dimethylarginine (ADMA), an endogenous inhibitor of nitric oxide synthase, is also directly affects VSMCs by influencing migration, apoptosis, and proliferation (Zhou et al., 2014). Studies have shown that miRNAs are involved in the detailed mechanism of how ox-LDL/ADMA induce VSMC phenotype change (Jin et al., 2020) (Sun et al., 2016). Of these, some clinical study have shown that dysregulation of circulating miR-182/miR-182–5p in patients with CAD or unprotected left main CAD by microarray profiling (Taurino et al., 2010) (Zhu et al., 2019). The expression of miR-182–5p was lower in the plasma of coronary atherosclerosis patients than in that of the healthy population. This approach may potentially provide a novel non-invasive diagnostic information and possible therapeutic targets in cardiovascular disease. miR-182–5p has been shown to inhibit the proliferation of vascular smooth muscle cells induced by ox-LDL by targeting pregnancy associated plasma protein A (PAPPA), further activating the NF-kB, PI3K/AKT and ERK signaling pathways (Jin et al., 2020). Furthermore, miR-182-3p was downregulated in ADMD-treated human aortic artery smooth muscle cells (SMCs) both *in vitro* and *in vivo*, and contributed to SMCs phenotype change by activating the ERK/MAP signaling pathway through upregulation of its direct target gene, myeloid-associated differentiation marker (MYADM) (Sun et al., 2016). Recently, researchers from the Peking Union Medical College have also described that the miR-182-3p/MYADM axis either inhibits pulmonary artery SMC proliferation via a KLF4/p21-dependent mechanism, or balances the BMP- and TGF-β signaling pathways in an SMC/EC-crosstalk-associated manner in pulmonary hypertension (Sun et al., 2020) (Bai et al., 2021). The ability of miR-182 in regulating multiple genes in signaling pathways makes it an attractive therapeutic target in AS and CAD.

### miR-182 in myocardial infarction and ischemia‒reperfusion injury

Acute myocardial infarction (AMI) and subsequent ischemia/reperfusion (I/R) injury are major causes of death and disability worldwide (Yellon and Hausenloy, 2007). Timely and effective myocardial reperfusion using either thrombolytic therapy or percutaneous coronary intervention (PCI) is an efficacious treatment in clinical practice (Neri et al., 2017). However, the process of reperfusion can induce myocardial reperfusion injury, which accounts for half of the final injury in AMI and contributes to many clinical complications (Moens et al., 2005) (Hausenloy et al., 2013). Currently, the exact pathophysiological mechanisms of I/R injury are not fully known (Simonis et al., 2012). Recently, a large experimental of evidence suggests that miRNAs are promising candidates to modulate the molecular and cellular processes involved in MI and I/R, including miR-182. Several groups have reported miR-182 targets implicated in cardiac apoptosis, including Nogo-C (neurite outgrowth inhibitor protein C) and CIAPIN1 (cytokine-induced apoptosis inhibitor 1). miR-182 levels were increased in the OGD/R (oxygen and glucose deprivation followed by reperfusion) model of H9c2 cells (Ren et al., 2019), but decreased by activating Nogo-C-mediated cell apoptosis (Jia et al., 2016). By targeting CIAPIN1, inhibition of miR-182–5p could also protect H9C2 cells from hypoxia-induced apoptosis (Zhang et al., 2018b). In addition, miR-182–5p has been shown to involved in the protective effects of ganoderic acid A (GAA) on H9c2 cells proliferation and apoptosis via regulating the mRNA and protein levels of PTEN and the PI3K/AKT signaling pathway (Zhang X. et al., 2018). In particular, transplantation of mesenchymal stromal cells (MSCs) or their exosomes has gradually become an attractive candidate for cardiac inflammation modulation. miR-182 could act as a master regulator of macrophage polarization *via* targeting TLR4 for a novel mechanism of the beneficial effect of MSCs-derived exosome transplantation after MI (Zhao et al., 2019).

In addition to MI, I/R injury is also associated with a broad array of life-threatening medical conditions including organ transplant. Recent studies have observed that I/R injury causes dysregulation of miRNAs during organ transplants. Rancan et al. showed that the expression of miR-182 significantly increased and contribute to pulmonary artery hypertension vascular remodeling *via* targeting MYADM to regulate KLF4/p21 signaling pathway after I/R in pigs with lung auto-transplantation (Rancan et al., 2016). miR-182 has also been shown to regulate hepatic ischemia‒reperfusion injury by activating SIRT1 through downregulation of its direct target gene, XBP1 (Li et al., 2021). A monocentric retrospective study of 38 patients with the anamnesis of acute cellular rejection (ACR) of heart allografts reflected that miR-182 was statistically significantly dysregulated in endomyocardial biopsies (with, prior to and after ACR) by Next-Generation Sequencing (Nováková et al., 2019). These findings demonstrated that a group of microRNAs were discovered to be changed in endomyocardial biopsies during I/R injury, and a diagnostic score based on their relative levels was developed for I/R injury diagnosis. miR-182 is known to regulate FOXO1 expression (Roser et al., 2018). Wei et al. performed microarray miRNA profiling in a murine model of cardiac allograft rejection and subsequently found that miR-182 increased in allograft tissues, graft-infiltrating mononuclear cells and plasmas (Wei et al., 2012). Moreover, in their subsequent study, CD4 + T cells were the main cellular source of miR-182 during allograft rejection, and miR-182^−/−^ mice showed augmented allograft survival *via* targeted modulation of FOXO1 expression (Wei et al., 2017). These studies suggest that miR-182 could also serve as a potential therapeutic target, which delineates new and strong mechanisms for patients following heart transplantation.

Furthermore, it is also becoming recognized that inhibiting or restoring specific miRNAs has the potential to regulate other non-coding RNAs. miR-182-5p may bind to the 3′-UTR of BNIP3 (Bcl-2/E1B 19 kDa-interacting protein 3) to repress its high expression after I/R, which prevents excessive cell death triggered by pathologic conditions (Jia et al., 2020) (Bellot et al., 2009). Deng et al. found that RT-qPCR analysis showed lncRNA SNHG14 was highly expressed and promoted OGD/R-induced neuronal injury by inducing excessive mitophagy *via* the miR-182-5p/BINP3 axis in HT22 cell cell model established by OGD/R (Deng et al., 2020). Of note, a small molecule inducer of miR-182, kenpaullone compound 5, suppressed cardiac cell death and improved heart function by downregulating BNIP3 in I/R-injury rats (Lee et al., 2016). Given these key roles and functions of miR-182 in cardiovascular disease, targeting miR-182 may regulate complex biological processes as a potential therapy for disease. However, issues such as immune stimulatory effects, mode of delivery and off-target effects pose a major challenge in bringing miRNA therapeutics into mainstream clinical practice. These concerns must be addressed in future studies.

### miR-182 in cerebrovascular diseases

There are many similar pathobiology and clinical manifestations between ischemic stroke and myocardial ischemia. Among the various underlying mechanisms of I/R injury, adequate regulation of miRNA levels may play a critical role in the prevention and treatment of I/R. In an observational clinical study, it was observed that patients with acute cerebral infarction and cerebral hemorrhage had significantly lower expression of miR-126 and higher expression of miR-182 when compared to subjects in the control group. Patients with mild conditions or good prognosis had higher miR-126 expression and lower miR-182 expression than patients with severe conditions or poor prognosis, and the combination of miR-126 and miR-182 showed better prognosis accuracy (Qi et al., 2020). The analysis of microRNA expression detected by microarray showed that the expression of miR-182 was downregulated in the rat cerebral cortex in left common carotid artery ligation models (Cui and Yang, 2013). A similar finding was also observed in BV2 cells with an OGD model, which showed miR-182-5p mimics attenuates cerebral I/R injury by targeting TLR4 (Wang et al., 2018a). Conversely, miR-182 was selectively upregulated after ischemic preconditioning in mice by transient occlusion of the middle cerebral artery (Lee et al., 2010), and stroke volume and neurological score were significantly improved by pre-treatment with miR-182 antagomir (Alhadidi et al., 2022). Additionally, the expression of miR-182 was found to increase after permanent middle cerebral artery occlusion (pMCAO), and it exacerbated cerebral ischemia injury by targeting inhibitory member of the ASPP family (iASPP) or aggravated blood-brain barrier disruption by downregulating the mTOR/FOXO1 pathway in cerebral ischemia (Yi et al., 2017) (Zhang et al., 2020). The inconsistency with miR-182 expression level in patients with cerebrovascular diseases suggesting further work needs to be done to establish a greater degree of accuracy on this matter. The protective effects of propofol (PPF) and exosomes released from astrocytes induced by the OGD/R model with berberine pretreatment (BBR-exos) have been reported in cerebral I/R injury, and the expression of miR-182 is upregulated (Hu et al., 2021) (Ding et al., 2023). The reason for the protective effect of PPF is that downregulated lncRNA MALAT1 increases miR-182 expression and suppresses the expression of TLR4, while the reason for the protective effect of BBR-exos is that highly expressed miR-182-5p in BBR-exos inhibits neuroinflammation by targeting Rac1. These findings suggest that targeting miR-182 could be a new therapeutic approach to address cerebrovascular diseases.

### miR-182 in cardiac hypertrophy and hypertension

Hypertension is one of the most prevalent chronic diseases (Mozaffarian et al., 2016). Maladaptive cardiac hypertrophy occurs as a consequence of persistent high blood pressure which is characterized by an enlargement of cardiomyocytes (CMs) and heart mass. Cardiac hypertrophy is a physiological response to physiological and pathological stimuli in hypertensive heart disease, MI, hypertrophic cardiomyopathy, and other CVDs. Persistent cardiac hypertrophy ultimately leads to eccentric heart dilation, HF and sudden death (Norton et al., 2002). For decades, a growing number of studies have suggested that previously unrecognized mechanisms, including non-coding RNAs, positively or negatively regulate cardiac hypertrophy and hypertension. The researchers discovered that hsa-miR-182-5p was associated with hypertension through the use of weighted gene co-expression network analysis (WGCNA), which helped to improve the understanding of the pathogenesis of hypertension (Li et al., 2020). Specifically, rno-miR-182 was upregulated and associated with remodeling in aging (12 months) spontaneously hypertensive rats (SHRs) when compared with young (3 months) SHRs (Wang et al., 2018b). miR-182 had a similar expression trend in myocardial hypertrophy associated with myocardial angiogenesis which was induced by placental growth factor (PlGF) in neonatal rat cardiomyocytes (NRCs) and mouse hearts. Their mechanism of action newly identified miR-182 target Bcat2 and regulate the expression of Akt/mTORC1 pathway (Li et al., 2016). Moreover, the expression of lncRNA SNHG16 was at a high level in the cardiac hypertrophic model induced by angiotensin II in neonatal mouse cardiomyocytes through repressed regulation of the miR-182-5p/IGF1 axis (Wang et al., 2020). New technologies such as cholesterol-containing nanocarriers, which can efficiently deliver inhibitor of miR-182 into hearts, have been developed. This nanosystem can significantly suppress cardiac hypertrophy in transverse aortic constriction (TAC) mice through miR-182 targeting FOXO3 and increasing its expression (Zhi et al., 2019).

### miR-182 in heart failure

Heart failure (HF) is considered an epidemic disease in the modern world and results in different, parallel developing clinical signs and symptoms. These signs and symptoms sum up to an unspecific clinical picture; thus it is urgent to explore invasive and non-invasive diagnostic tools to obtain accurate diagnosis, treatment and prognosis (Tanai and Frantz, 2015). miR-182 was shown to be upregulated in the sera of HF patients (stable, acute decompensated), and which may be a potential prognostic marker by ROC analysis and Cox regression analysis (Cakmak et al., 2015) (Zhirov et al., 2019). miR-182 was found to be inversely correlated with left ventricular mass index in symptomatic HF patients with systolic dysfunction (Ikitimur et al., 2015). Previous studies indicated that a low circulating BDNF (brain-derived neurotrophic factor) level was linked with a poor prognosis in cardiovascular diseases (Montone et al., 2021) (Yılmaz, 2019). A recent study showed a novel correlation between BDNF and miR-182-5 have been described in HF patients. The expression of miR-182-5p was upregulated and the BDNF level was decreased in HF patients. Moreover, miR-182-5p combined with BDNF predicted prognosis for better prevention in HF by identifying and following risk groups (Fang et al., 2022). These clinical findings suggest that down-regulating the expression level of miR-182 may help improve ventricular remodeling and prognosis in patients with chronic HF, but further study is required for validation.

### miR-182 in congenital heart disease and cardiotoxicity

Congenital heart disease (CHD) is one of the most common birth defects (Lage et al., 2012) (Diab et al., 2021). Genetic or chromosomal abnormalities, epigenetic factors, excessive alcohol consumption during pregnancy, the use of medications, and maternal viral infection in the first trimester of pregnancy are all risk factors for congenital heart disease in children (Diab et al., 2021). CHD is the leading non-infectious cause of mortality in newborns, especially cyanotic CHD (Rohit and Rajan, 2020). miR-182 was shown to be decreased in serum samples from patients with cyanotic CHD and overexpression of miR-182 in hypoxia-induced H9c2 cells that was associated with promoted cell proliferation and decreased apoptosis through targeting HES1 (Zhang et al., 2018c). Trichloroethylene (TCE) is a common environmental pollutant that is associated with congenital cardiac defects. TCE exposure induced heart defects and dysfunctions, and upregulated miR-182 in the hearts of zebrafish embryos (Huang et al., 2020). In addition, miR-182-5p was upregulated in human-induced pluripotent stem cell-derived cardiomyocytes (hiPSC-CMs) that were exposed to doxorubicin, etoposide or kinase inhibitors (Chaudhari et al., 2016) (Nemade et al., 2018) (Gryshkova et al., 2022). These observations provide new evidence for an unexpected and sensitive role of miR-182 as a cardiotoxicity biomarker for screening novel drugs and environmental cardiotoxicity.

## Conclusion

Dysregulation of miR-182 contributes to the development of a variety of cardiovascular and cerebrovascular diseases, including atherosclerosis, CAD, MI, I/R injury, organ transplantation, cardiac hypertrophy, hypertension, heart failure, congenital heart disease and cardiotoxicity (Table 1). New insights into the involvement of miR-182 in the regulation of proliferation, migration, hypoxia, ischemia, hypertrophy and apoptosis have provided alternative ways to inhibit cardiac pathology with the potential for translation into the clinic. However, the same miR-182 members play different roles in different diseases and pathological processes. So, further researches on miR-182 in cardiovascular and cerebrovascular diseases are needed. Targeting miR-182 function with inhibitors or mimics has become a viable option for the modulation of proteins and other miRNAs that are dysregulated in cardiovascular and cerebrovascular diseases. However, the therapeutic application of miR-182 in cardiovascular diseases is still in its infancy. Most studies have only observed changes in the expression levels of miR-182 in the serum of CVD patients, mainly focusing on CAD and CHF patients, and there is a lack of relevant studies on the further intervention of miR-182. What’s more, some miR-182 family members have two sides in the role of cardiovascular and cerebrovascular diseases. For example, inducing miR-182 expression by kenpaullone compound 5 can suppress cardiac cell death and improved heart function (Lee et al., 2016). Nevertheless, miR-182 is also a regulator of cardiac hypertrophy. New nano system which can efficiently deliver inhibitor of miR-182 into hearts can significantly suppress cardiac hypertrophy (Zhi et al., 2019). Therefore, it is essential to identify the optimal balance between their harmful and beneficial effects to maximize their therapeutic potential while minimizing their negative impact. In addition, there have been few studies on the delivery system of miR-182, and it is necessary to find a suitable miR-182 vector that can target our specific cells for precision treatment. Meanwhile, there is a lack of studies on long-term effects. Current studies mostly focus on the short-term effects of miRNA, and there is not enough evidence to prove that miRNA can have a sustained therapeutic effect on CVD. Optimizing the stability of miR-182, improving delivery systems, controlling off-target effects and targeted drug delivery remain hurdles that need to be overcome for future development of therapeutic applications of miR-182 in CVD. Based on the aforementioned limitations, in order to provide new preventive and therapeutic targets for the treatment of cardiovascular and cerebrovascular diseases, more clinical trials translating animal experimental results need to be evaluated in future studies to evaluate the mechanism of the action of miR-182.
